# The proportion of diabetic ketoacidosis emergencies during Ramadan fasting: a retrospective study

**DOI:** 10.3389/fendo.2026.1895364

**Published:** 2026-08-31

**Authors:** Maram H. Alsufyani, Areej Y. Hamdi, Nawal K. Alanazi, Reema Alalmaee, Gadah Aljarallah, Rahaf Alsufyani, Ahmed Alsumaily

**Affiliations:** 1 Department of Primary Health Care, Ministry of National Guard - Health Affairs, Taif, Saudi Arabia; 2 Department of Endocrinology and Diabetes, Ministry of National Guard - Health Affairs, Riyadh, Saudi Arabia; 3 King Abdullah International Medical Research Center, Riyadh, Saudi Arabia; 4 Diabetes and Endocrinology Center of King Fahad Central Hospital, Ministry of Health, Jazan, Saudi Arabia; 5 Diabetes and Endocrinology Center of King Fahad Specialist Hospital, Ministry of Health, Tabuk, Saudi Arabia; 6 Department of Medicine, King Abdulaziz Medical City, Ministry of National Guard Health Affairs, Riyadh, Saudi Arabia; 7 College of Medicine, Taif university, Taif, Saudi Arabia; 8 King Saud bin Abdulaziz University for Health Sciences, Riyadh, Saudi Arabia

**Keywords:** correlates, diabetic emergency, diabetic ketoacidosis, hyperglycemia, Ramadan

## Abstract

**Objectives:**

Ramadan fasting is an annual religious event in the Muslim calendar that has been shown to affect diabetes control. The relationship between Ramadan fasting and increased diabetic ketoacidosis (DKA) presentations was studied in several settings, but results are still mixed. Our study aims to address this knowledge gap and estimate the frequency and correlates of DKA emergencies during the month of Ramadan using a more informative study design with repeated observations over three months during the 3-year study period.

**Methods:**

This was a retrospective, medical record-based observational 3-year study with repeated observations over the 3-month period of pre-Ramadan, Ramadan, and post-Ramadan.

**Results:**

This study included a total of 125 patients with an established diagnosis of DKA who presented to the hospital during the 3-year study period. Of these, 56 (44.8%) patients were diagnosed with DKA during Ramadan, far in excess of the number diagnosed during the other months, by a 1.75 ratio for Sha’aban and a 1.51 ratio for Shawwal. The severity of DKA presentations was far less during Ramadan than during the other months (odds reduced by 47.4%, p = 0.01803). In addition, older age was associated with a more severe presentation (odds increased by 4.7% for each additional year of age, p = 0.00885).

**Conclusions:**

Consistent with the literature, we found that severe DKA presentations during Ramadan were more likely to occur in older adults. We also found that when DKA was diagnosed during Ramadan, it was less likely to be severe than presentations during both the Sha’aban and Shawwal months combined. This, we theorize, may be explained by the higher carbohydrate dietary content during Iftar (breakfast) and Sahoor (pre-fasting meal). This is not a well-established fact, however, and should await studies that focus on the association between nutritional intake during these special times and the severity of DKA presentations.

## 1 Introduction

Ramadan is the ninth month of the Hijri calendar in Islam. It is one of the holy months in Islam, during which a 30-day period of mandatory fasting takes place. During the fast, Muslims refrain from eating, drinking, and using oral medications between dawn and sunset. Fasting during Ramadan has several physiological effects on a range of homeostatic and endocrine processes (1). For instance, diabetes control is directly impacted by Ramadan fasting, owing to the lengthy period of fasting and the substantial changes in mealtimes, types of foods, use of medication, and daily lifestyle (2). During Ramadan fasting, eating and drinking are entirely nocturnal. A fasting person typically has two meals: a large, high-calorie meal after sunset (Iftar) and a small, low-calorie meal just before dawn (Sahoor). Many snacks may be consumed between these two main meals. Meals during Ramadan nights are characteristically high in carbohydrates and fat content (3). Patients living with diabetes are exempt from fasting during Ramadan; however, a significant number will choose to fast at times despite advice from their family physician or treating diabetologist (4, 5). Fasting during Ramadan among people with diabetes is associated with significant risks that have prompted clinicians to attempt and quantify person-centered scores that may assist patients in making a risk-informed decision about whether to fast (6). When people with diabetes fast and maintain long hours without food and water, blood glucose levels may become elevated, and when insulin is unable to facilitate the use of glucose for energy, the body may run out of energy and begin to break down fat cells, producing ketones and making the blood acidic, leading to diabetic ketoacidosis (DKA). Such hormonal instability may also result in conditions such as hyperglycemia, lipolysis, and DKA (7). Fasting and dehydration have different mechanisms affecting glycemia during insulin omission. Dehydration favors the development of hyperglycemia, whereas fasting causes reduced glucose concentrations. This finding probably accelerates the development of lipolysis and ketosis and increases glucagon levels, in addition to causing modifications in substrate availability and counterregulatory hormone concentrations. The severity of fasting and dehydration likely clarifies much of the variability in plasma glucose concentrations observed in diabetic ketoacidosis (8). The triad of uncontrolled hyperglycemia, metabolic acidosis, and increased total body ketone concentration is considered the diagnostic criteria of diabetic ketoacidosis. These metabolic instabilities primarily result from the combination of complete or partial insulin deficiency and an increase in counterregulatory hormones (glucagon, catecholamines, cortisol, and growth hormone). The majority of patients with DKA have insulin-dependent autoimmune type 1 diabetes. The diagnostic criteria used to diagnose DKA in our study were the same as those mentioned in the American Diabetes Association guidelines:

1. The plasma glucose concentration was ≥ 200 mg/dL or there was a prior history of diabetes.
2. PH <7.30 or serum bicarbonate < 18 mEq/L.
3. There was a presence of ketonuria (2+ or more) or serum ketones were ≥ 3.0 mmol/L.

Diabetic ketoacidosis severity is defined as follows:

1. Mild DKA is categorized by a pH level of 7.25–7.3 and serum bicarbonate of 15–18 meq/L.
2. Moderate DKA is categorized by a pH level of 7.0–7.24 and serum bicarbonate of 10–15 meq/L.
3. Severe DKA is categorized by a pH of <7.0 and a serum bicarbonate level of <10 meq/L (9).

We noted that many studies were conducted in Saudi Arabia and the wider Gulf and Middle East regions to address the incidence of diabetic ketoacidosis during Ramadan fasting. A retrospective study of all adults hospitalized by CKK at King Saud Medical City in Riyadh (10) found a significant increase in DKA presentations during Ramadan (due to missed insulin doses) compared to Sha’aban, and a further, larger increase in Shawwal (when infections were the main cause of DKA). However, one retrospective cross-sectional study conducted at the National Guard Hospital in Jeddah found that hyperglycemia among patients with type 2 diabetes and insulin-treated patients was recorded as the most common feature of diabetes emergency visits during the three months studied, with no significant differences between the months (11). One recent UAE study confirmed that the number of DKA hospitalizations during Ramadan was not significantly higher than the average monthly number of hospitalizations over a 10-year period. This seasonality was not associated with the month of Ramadan (12). Another study conducted in three major hospitals in the United Arab Emirates indicated that DKA presentations in Ramadan were associated with a higher frequency of urinary tract infection and a longer hospital stay, although glycemic control was not worse than in other months (13). These results were comparable to those of a similar but larger-scale study that showed that the duration of acidosis was longest during the Ramadan month (14). A Libyan investigation found that there was no increase in the incidence and mortality from DKA during Ramadan, which might indicate that Ramadan fasting is not a significant risk factor for diabetic ketoacidosis (15). Furthermore, a Malaysian study found that fasting during Ramadan does not lead to an increase in admissions due to diabetes emergencies (16). Therefore, meta-analysis-based recommendations indicated that, with appropriate education, fasting during Ramadan is possible for patients with insulin-dependent diabetes (17).

The primary aim of the current study was to estimate the frequency of diabetic ketoacidosis emergency presentations during Ramadan in comparison to the month before (Sha’aban) and the month after (Shawwal) in the setting of emergency care in Saudi Arabia.

We also aimed to estimate the frequency of diabetic ketoacidosis emergency presentations during the pre-Ramadan, Ramadan, and post-Ramadan months among patients attending the emergency department of King Abdulaziz Medical City-Riyadh over the last 3-year period from 2021 to 2023. Furthermore, the current investigation aimed to explore predictors of diabetic ketoacidosis during Ramadan from 2021 to 2023. Moreover, we aimed to assess the severity of diabetic ketoacidosis during Ramadan from 2021 to 2023 and to explore major differences between Ramadan and the other months from 2021 to 2023.

## 2 Materials and methods

### 2.1 Study area

This was a retrospective study conducted through a review of the medical records and the health information system ‘BESTCare’ of all adult patients with diabetic ketoacidosis who visited the emergency room (ER) department of King Abdulaziz Medical City, Ministry of National Guards – Health Affairs, Riyadh city, Saudi Arabia, during the Sha’aban, Ramadan, and Shawwal months over the past 3 years. The age of 14 years was chosen as the minimum age for inclusion in the study.

### 2.2 Study participants

Inclusion criteria: all patients 14 years of age or older who presented to the ER with a confirmed diagnosis of diabetic ketoacidosis and were known to have type 1 diabetes mellitus (T1DM). Exclusion criteria: pregnant women and patients younger than 14 years or older than 60 years were excluded from the study to avoid inclusion of patients with gestational diabetes and pediatric patients. We also excluded patients with type 2 diabetes.

### 2.3 Study design

Retrospective, medical record-based observational study.

### 2.4 Sample size

We aimed to conduct a complete enumeration of patients who visited the ER and met the inclusion criteria during Sha’aban, Ramadan, and Shawwal over the period from 2021 to 2023. All patients who attended the ER during the study period and met the inclusion/exclusion criteria were included in the final investigation.

### 2.5 Sampling technique

The medical files of all patients who met the inclusion criteria were reviewed during the period from 2021 to 2023 after confirmation of the diagnosis of diabetic ketoacidosis.

### 2.6 Data collection methods, instruments used, and measurements

Data were collected on the following variables: patient age, sex, type of diabetes, duration of diabetes, HgA1c level in the last 6 months, precipitating factors, severity level, duration of symptoms before presentation to the ER, length of hospitalization, and biochemical findings at presentation, including plasma glucose level, presence of ketones in urine, venous blood pH, and bicarbonate (HCO3). Serum potassium and sodium levels were also recorded. In addition, vital signs (pulse rate, respiratory rate, systolic and diastolic blood pressure, temperature, and O2 saturation) were recorded. The diagnostic criteria used to diagnose DKA in our study were as follows. We adopted the American Diabetes Association guidelines: plasma glucose concentration ≥ 200 mg/dL or a prior history of diabetes, pH of <7.30 or serum bicarbonate of 18 mEq/L, and presence of ketonuria (2+ or more) or serum ketones ≥3.0 mmol/L. The severity of DKA was defined as follows: mild DKA was categorized by a pH level of 7.25–7.3 and a serum bicarbonate level of 15–18 mEq/L; moderate DKA was categorized by a pH of 7.0–7.24 and a serum bicarbonate level of 10–15 mEq/L; and severe DKA was defined by a pH of <7.0 and a serum bicarbonate level of <10 mEq/L (9).

### 2.7 Data management and analysis plan

The data entries were checked for completeness, and responses were coded and entered into the Statistical Package for the Social Sciences (SPSS) software, version 25 for Windows. The results were then tabulated and graphically and statistically analyzed SPSS. Normally distributed data, such as duration of diabetes in months, were expressed as the mean ± standard deviation, whereas variables not normally distributed, such as age in years, were presented as the median and interquartile range. Comparisons between qualitative variables were made by using Fisher’s exact test or the chi-square test. Data with a normal distribution were compared using Student’s t-test and analysis of variance (ANOVA). Comparisons of continuous variables with an asymmetric distribution were made using the Mann–Whitney U test and the Kruskal–Wallis test. Associations between variables were explored using Pearson’s correlation and Spearman’s rho (for data that were not normally distributed). A binomial regression model was applied to identify significant associations between specific factors and the development of diabetic ketoacidosis. Other statistical tests were applied as appropriate. A p-value of 0.05 was used to determine statistical significance.

### 2.8 Ethical considerations

The study was approved by the King Abdulla International Medical Research Center Ethics Committee (approval number RYD-23-419812-142672, protocol number NRC23R/513/08).

## 3 Results

### 3.1 Sociodemographic correlates

This study included a total of 125 patients with an established diagnosis of DKA who presented to the hospital during the study period. Of these, 56 (44.8%) were men and 69 (55.2%) were women. The mean age was 25.3 years, ranging between 15 and 55 years. The median age was 23 years. Age was not normally distributed (Shapiro–Wilk W = 0.89829, p < 0.0001). .

There were 12 (9.6%) patients with newly diagnosed diabetes. The mean duration of diabetes in the sample was 117.6 months (SD = 78.7 months). The duration of diabetes, as shown in **Table 1**, ranged between 0 months (newly diagnosed) and 300 months (25 years). The median duration was 120 months (10 years). The duration of diabetes was non-normally distributed (Shapiro–Wilk W = 0.95843, p = 0.0022).

**Table 1:** Age, sex, and duration of diabetes distribution.

Factor	Count (%)/ mean (SD)	Shaban	Ramadan	Shawwal	Significance
Sex					
Male Female	56 (44.8%) 69 (55.2%)	13 (40.6%) 19 (59.4%)	24 (42.9%) 32 (57.1%)	19 (51.4%) 18 (48.6%)	Chi-square = 0.9532 P = 0.6209
Age	Mean = 25.3 years (SD = 8.4 years)	Mean = 25 years (SD = 9.1 years)	Mean = 26.4 years (SD = 8.9 years)	Mean = 24 years (SD = 7.1 years)	Kruskal–Wallis = 1.767 p = 0.4133
Duration of diabetes	Mean = 117.6 months (SD = 78.7 months)	Mean = 93.4 months (SD = 62.1 months)	Mean = 122.1 months (SD = 83 months)	Mean = 124.8 months (SD = 76.3 months)	Kruskal–Wallis = 1.7187 p = 0.4234

In terms of the frequency of precipitating factors of DKA, the most common precipitating factor was non-compliance with medications, accounting for 98 (78.4%) of all presentations.

In terms of DKA severity, there were 57 (45.6%) severe DKA cases, compared with 44 (35.2%) moderate DKA presentations and 24 (19.2%) mild DKA cases during the study period. The majority of patients, 83 (66.4%), reported a symptom duration of 1 day, followed by 25 (20%) whose symptom duration was 2 days and 10 (8%) who had experienced symptoms for 3 days before presenting to the emergency department. See Supplementary Materials 1 and 2.

The mean duration of acidosis until correction was 17 h (SD = 8.6 hours), ranging from a minimum duration of 3 h to a maximum of 47 h. The median duration was 15 h.

**Figure 1:**
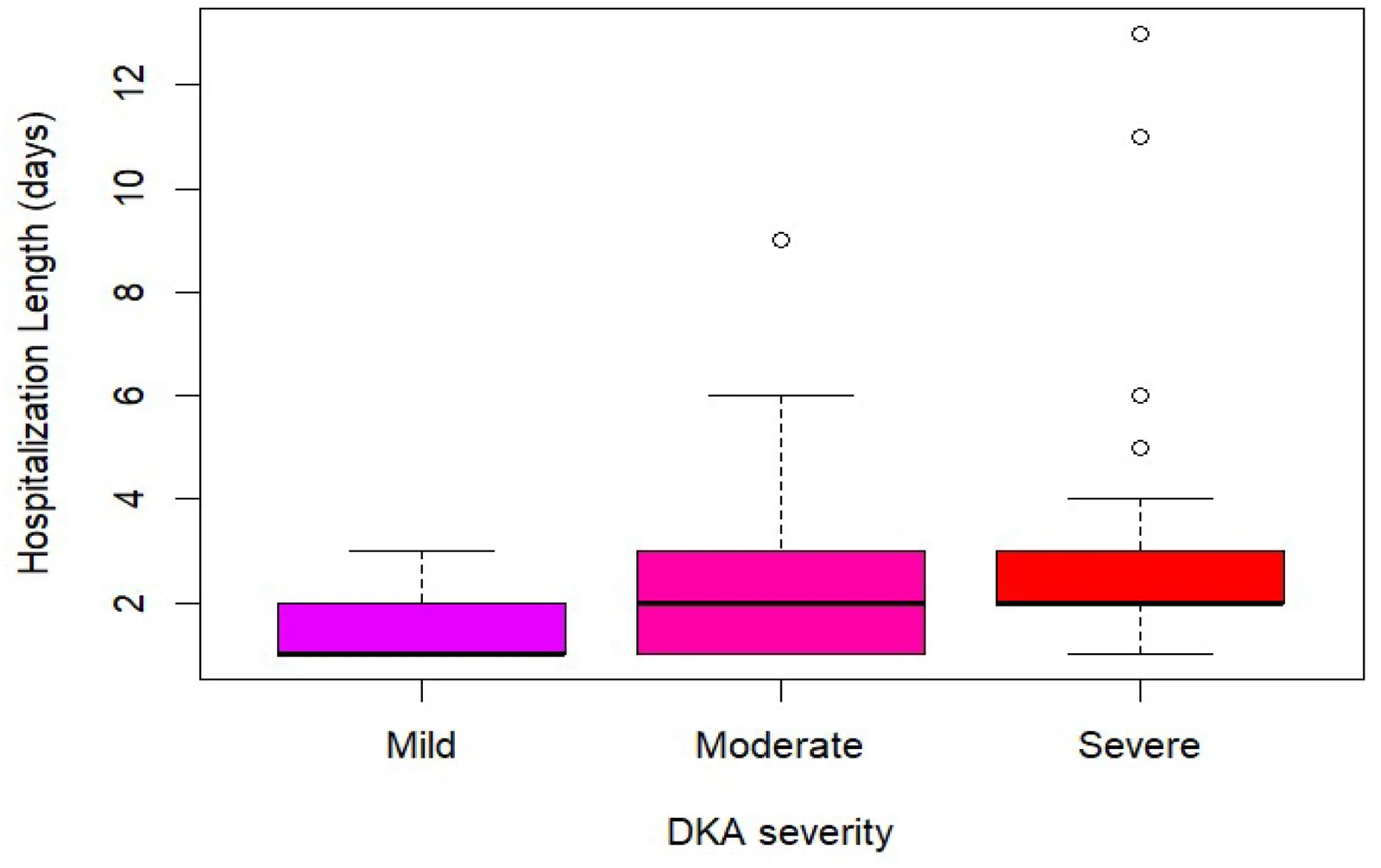
Box-and-whisker plots showing the correlation between length of hospitalization and DKA severity (p = 0.0002559).

The mean length of hospitalization was 2.3 days (SD = 1.84 days, median length of hospitalization = 2 days), ranging from 1 day to 13 days in total. Notably, the majority of patients were discharged within 3 days of admission. In all, 44 (35.2%) patients were discharged within a single day, 42 (33.6%) were discharged by the second day, and 24 (19.2%) were discharged on the third day (**Figure 1**).

It was notable that the severity of DKA presentation correlated with the length of hospitalization. The mean duration of hospitalization for patients with the mild presentation was 1.4 days, which increased to 2.3 days among patients with moderate DKA and to 2.7 days among those with severe DKA. This difference was statistically significant, Kruskal–Wallis chi-square = 16.541 (DF = 2), p = 0.0002559.

It was notable that the majority of patients (n = 105, 84%) had no pre-Ramadan diabetes health education, whereas only 17 (13.6%) had received such health education. It was also notable that 9 (52.9%) of those who received health education presented with severe DKA, compared with 5 (29.4%) who presented with moderate DKA and 3 (17.6%) who had mild DKA.

In terms of laboratory and diabetological indices, see **Table 2** below.

**Table 2:** Laboratory and diabetological indices.

Diabetological index	Mean (SD)	Minimum	Maximum	Median
HbA1c	10.97 (2.28)	6	17	11
Plasma glucose	25.9 (8.55)	4	44	26
Urinary ketones	126.5 (42.18)	10	300	150
Venous pH	7.152 (0.111)	6.873	7.35	7.185
Bicarbonate	11.2 (5.20)	3	27	10
Potassium	5.0(1.20)	3.0	13.0	5.0
Pulse	115.3 (16.7)	75	163	116
Respiratory rate	21.5 (4.5)	14	42	20
Systolic BP	122 (16.6)	80	188	120
Diastolic BP	72 (12.6)	44	110	71
Temperature	36.95 (0.25)	36	38	37
O2 saturation	98.4 (1.43)	93	100	99
BMI	23.8 (5.55)	13	44	23

### 3.2 Proportion of DKA presentations during Ramadan

This study aimed to estimate the frequency of diabetic ketoacidosis presentations during Ramadan in comparison to the month before (Sha’aban) and the month after (Shawwal) in the setting of emergency care in Saudi Arabia.

In terms of DKA presentations during Ramadan, 56 (44.8%) patients were diagnosed with DKA during Ramadan. This compared with 32 (25.6%) similar presentations in Sha’aban (the month prior to Ramadan) and 37 (29.6%) presentations in Shawwal (the month after Ramadan). Ramadan presentations were substantially more frequent than those in the other months, with a 1.75 ratio compared with Sha’aban and a 1.51 ratio compared with Shawwal.

The severity of DKA cases varied among the three months studied. Among all presentations in the Shaaban month, severe DKA cases constituted 20 (62.5%) of presentations. In comparison, 23 (41.1%) of all Ramadan presentations were severe, compared to 14 (37.8%) of all Shawwal presentations. See **Table 3** below.

**Table 3:** Severity of DKA presentation according to Hijri month.

DKA severity	Shaaban, count (%)	Ramadan, count (%)	Shawwal, count (%)
Mild	2 (6.25%)	16 (28.8%)	6 (16.2%)
Moderate	10 (31.25%)	17 (30.4%)	17 (45.9%)
Severe	20 (62.5%)	23 (41.1%)	14 (37.8%)
Total	32	56	37

**Figure 2:**
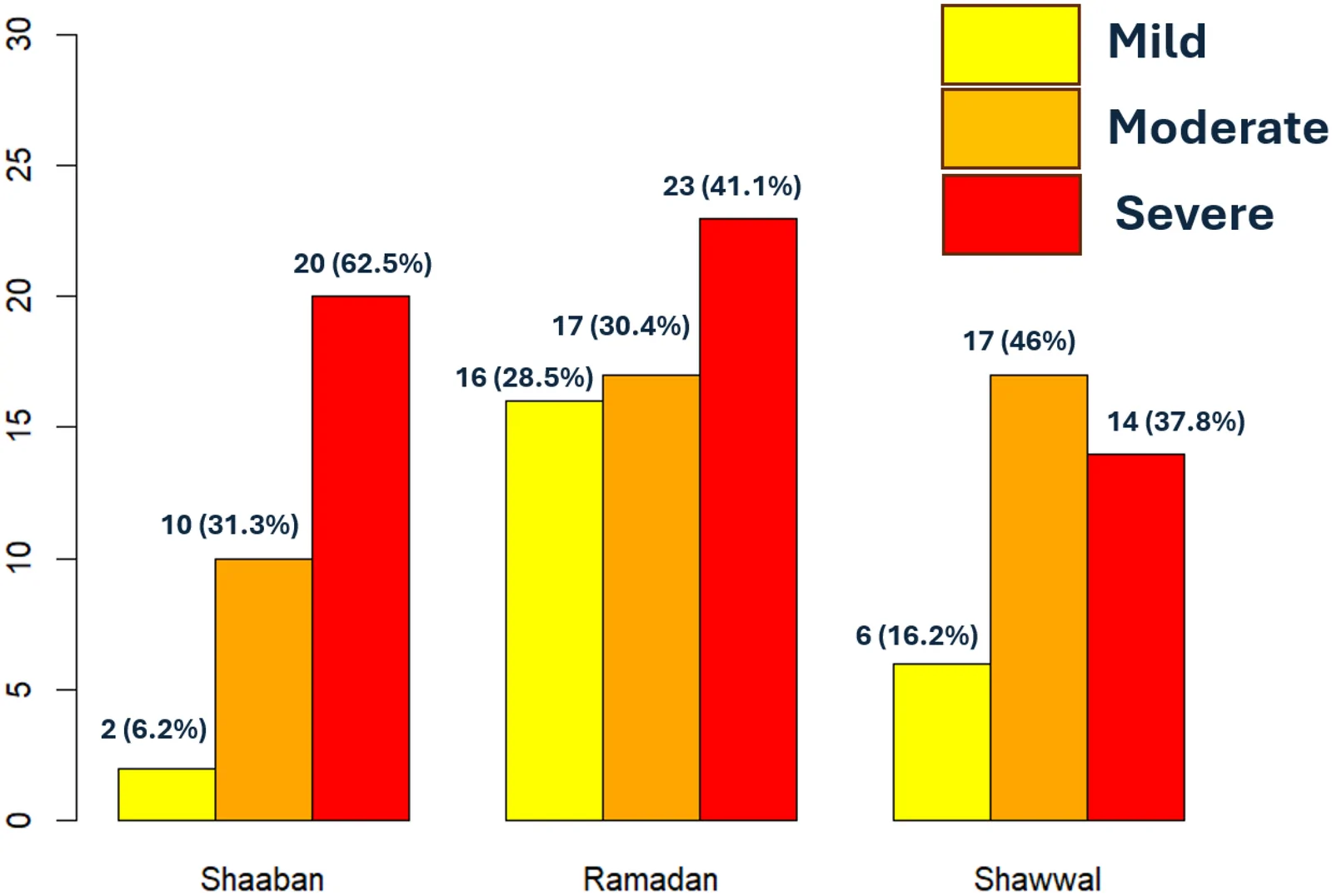
Stacked bar plots showing the correlation between month of presentation and DKA severity (p = 0.0404).

The unadjusted difference in terms of the excess of severe presentations in Sha’aban, compared with the excess of moderate presentations in Shawwal and mild presentations in Ramadan, was statistically significant (chi-square = 10.002, DF = 4, p = 0.0404). See **Figure 2** below.

**Figure 3:**
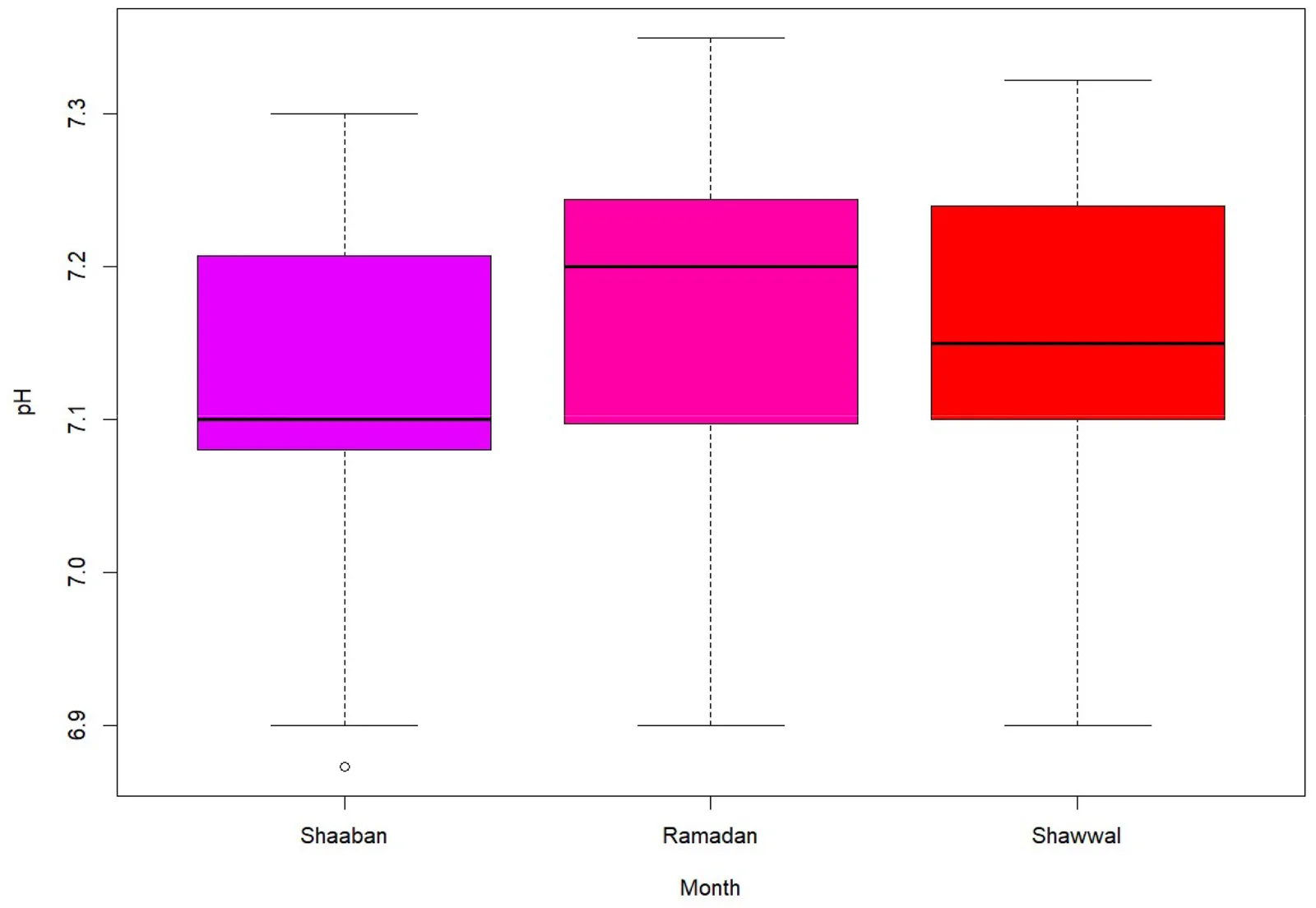
Box-and-whisker plots showing the correlation between pH and month of DKA presentation (p = 0.07079).

In terms of predictors of diabetic ketoacidosis during Ramadan from 2021 to 2023, as displayed in **Figure 3**, we calculated the median pH among patients presenting in different months. Ramadan presentations had the highest median pH of 7.20, followed by Shawwal presentations (median pH = 7.15) and Sha’aban presentations (pH = 7.10); however, this difference was not statistically significant (Kruskal–Wallis chi-square = 5.2962, DF = 2, p = 0.07079).

### 3.3 Adjusted binomial modeling of predictors

To further study the effect of demographic, diabetological, and clinical predictor variables on DKA presentations during Ramadan, we modeled the data using a full, saturated binomial regression analysis, with the severity of presentation (mild, moderate, or severe) as the predicted variable. This analysis focused on the specific and unique attributes of patients presenting with DKA during Ramadan.

**Figure 4:**
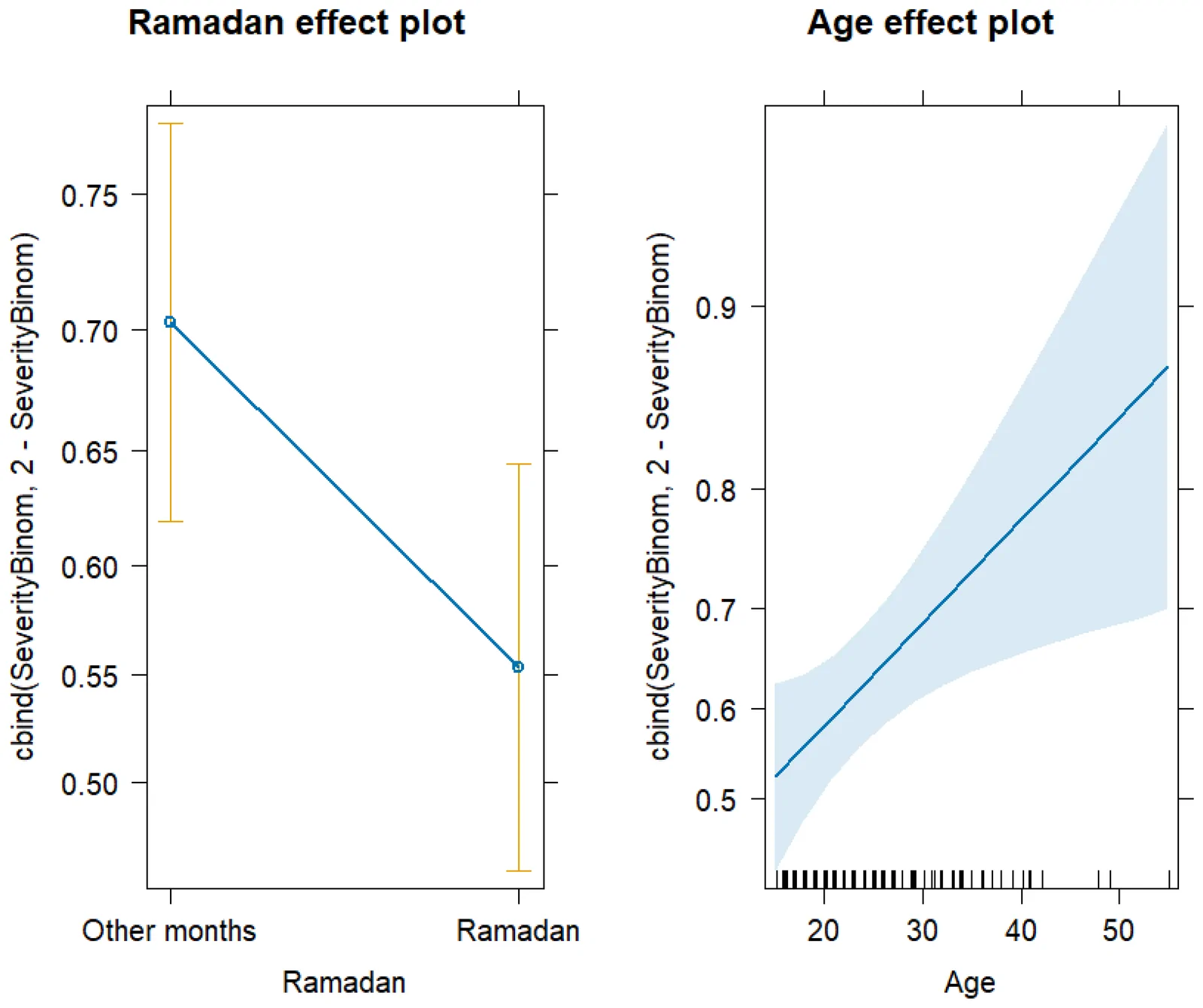
Binomial regression effects of Ramadan presentation and age on DKA severity presentation.

Only two variables significantly predicted the severity of DKA presentations: Ramadan month and age. See **Table 4** and **Figure 4**.

**Table 4:** Binomial regression effects of Ramadan presentation and age on DKA severity.

Factor	Odds	95% CI (odds)	β	SE	p value
Ramadan	0.5256	0.3084 to 0.8957	-0.6433	0.2720	0.01803
Age	1.0468	1.0116 to 1.0833	0.0458	0.01748	0.00885

The odds of a more severe DKA presentation were 47.4% lower during Ramadan than during the other months (p = 0.01803). In addition, older age was associated with a more severe presentation, with the odds increasing by 4.7% for each additional year of age (p = 0.00885).

## 4 Discussion

This current study encompassed data from a 3-year consecutive period with repeated 3-month observations of presentation to the ER by patients with DKA during the pre-Ramadan, Ramadan, and post-Ramadan periods. We have examined 125 hospital records for correlates and predictors of DKA presentations. Furthermore, we contrasted Ramadan presentations with non-Ramadan presentations to capture any salient differences. Such differences can be incorporated into the much-needed advice for patients with diabetes regarding current research recommendations on fasting and its associated risks during the holy month.

Our findings indicate an excess of Ramadan-related DKA cases, with an increase of approximately 75% compared with Sha’aban and 50% compared with Shawwal. Indeed, a recent study of the Ramadan-related risk found no increase in DKA presentations during the holy month (18). Other longitudinal studies concluded over approximately a 10-year-period concluded that DKA episodes were almost the same across all calendar months, including Ramadan (12). However, what is unique about our current investigation is the focus on the month preceding Ramadan and the month following Ramadan, rather than comparing Ramadan presentation with those occurring throughout the entire year. On the other hand, our results align well with very recent findings that DKA cases have increased incrementally during Ramadan season, with a further increase in presentations in Shawwal as well (10).

One of the most important findings from our analysis is that the severity of DKA emergency symptoms tends to be associated with older patients. This association between DKA severity and older age is an important finding in our study that seems to be under recognized in the literature. The bulk of diabetology research has focused on pediatric DKA presentations in terms of the association between age and symptom severity. A handful of studies among adults with DKA presentations found that greater symptom severity was associated with longer hospital stays (19). Exploratory studies confirmed that older age is associated with medical comorbidities, reduced renal function, and unfavorable physiological parameters that contribute to severe DKA presentations among older adults (20). The association between age and severity of DKA presentation was further shown to be a detrimental factor in increasing mortality, particularly in developing nations (21). Our study did not examine co-occurring medical conditions that could have worsened glycemic indices among our sample; however, this remains a ripe area for future research.

In addition, we found that the severity of DKA during Ramadan was less likely to be severe or moderate than that of presentations during both Sha’aban and Shawwal combined. This was an unexpected finding. Recent estimates for rates of severe DKA outside Ramadan in Saudi Arabia ranged between 30.8% and 61.6% of all presentations (22–24). In the United Arab Emirates, rates of severe DKA were estimated at 17.7%, particularly among newly diagnosed patients and patients with shorter duration of diabetes (25). However, according to our results, severe DKA proportions were 62.5% in Sha’aban, 41.1% in Ramadan, and 37.8% in Shawwal. It is notable that severe DKA proportions in Sha’aban were roughly at the higher end of the reported Saudi DKA rates. Yet, these severe DKA rates dropped during Ramadan and further into Shawwal. It is difficult, based on our data, to explore the underlying factors for such a drop. Further focused research should investigate whether such differences relate to higher rates of non-compliance in Sha’aban or the neglect of diabetes requirements in the period preceding Ramadan.

Pre-Ramadan health education was not associated with a significant difference in DKA presentations during Ramadan. This finding may indicate that the educational content, delivery, or timing may require improvement rather than that health education interventions are ineffective. Many educational packages have been shown to help reduce the frequency of DKA among patients with diabetes (26). One brief, Ramadan-focused educational intervention was provided to patients intending to fast and resulted in a less than 5% rate of DKA, but its overall impact on patients wellbeing remains to be explored through large-scale interventions (27). Educational content focusing on dietary control and continuous monitoring has recently shown a favorable impact on glycemic control among patients with type 1 diabetes (28). Patients with diabetes were found to be particularly concerned about improving their knowledge and understanding of fasting during Ramadan, along with their overall self-efficacy and well-being and coping with the challenges posed by Ramadan fasting to their health (29).

### 4.1 Strengths and limitations

Our study was essentially a 3-year observational exploration, with a design that avoided the cross-sectional nature common to many past studies carried out in the region. We recruited 125 patients, representing a modest sample size compared with past research papers. Notably, the sample size of 125 over 3 years is small, and the comparison groups are unequal (56 Ramadan vs. 32 Sha’aban vs. 37 Shawwal). Caution is required in interpreting the results, as the unequal distribution could reflect ascertainment bias, seasonal emergency room attendance patterns, or simply natural variation in a small dataset. A rate-based comparison (DKA presentations per total ER visits per month) would have been far more informative. We examined a large number of demographic and diabetological variables in addition to health indices. However, a number of limitations should be considered, as the results may not be fully generalizable to non-similar settings. The study design we have applied is subject to different limitations, such as information bias due to incomplete or inaccurate documentation, selection bias due to the inclusion of only patients with complete records, and uncontrolled confounding factors. Generally, the retrospective design and the 3-year study period may have influenced the consistency of the collected data, and the findings may have limited generalizability. We have not included other diabetic emergencies or non-diabetic presentations in our calculations; hence, the crude numbers of patients were not compared with the population of patients who presented during the same period. Nonetheless, we arrived at important results that could be translated into meaningful recommendations.

### 4.2 Future research directions

Further studies should address research gaps uncovered by our current study. These include comparing patients with diabetes who fast with those who do not fast during Ramadan, preferably on a day-by-day basis, to examine diabetological indices and health quality. A focus should also shift to quality of life during Ramadan and the sense of otherness experienced by patients with diabetes as cultural and religious routines change during the holy month.

Further research should specifically focus on patients who choose to fast during Ramadan and follow them closely for the development of diabetes emergencies in the short term and diabetes complications in the long term. Moreover, Ramadan tends to move between summer and winter months. Hence, a substantial research gap exists regarding the differential effects of this seasonal shift on diabetes emergencies and complications.

Further focused research should investigate whether such differences relate to higher rates of non-compliance in Sha’aban or the notorious negligence of diabetes requirements in the period preceding Ramadan.

Pre-Ramadan health education should be explored in large-scale educational and clinical interventions in terms of content, delivery, and timing (preferably prior to Sha’aban).

## 5 Conclusion

Patients with diabetes present more frequently to the emergency department with DKA during Ramadan; however, their presentations tend to be less severe in terms of symptomatology than those during other months. Yet, glucose levels were significantly higher among Ramadan DKA presentations.

## Data Availability

The raw data supporting the conclusions of this article will be made available by the authors, without undue reservation.

## References

[1] Hassanein M Afandi B Ahmedani MY Alamoudi RM Alawadi F Bajaj HS . Diabetes and Ramadan: practical guidelines 2021. Diabetes Res Clin Pract. (2022) 185:109185. doi: 10.1016/j.diabres.2020.109185 35016991

[2] Qureshi B . Diabetes in ramadan. J R Soc Med. (2002) 95:489–90. doi: 10.1177/014107680209501003 12356968 PMC1279173

[3] Fazel M . Medical implications of controlled fasting. J R Soc Med. (1998) 91:260–3. doi: 10.1177/014107689809100505 9764079 PMC1296701

[4] Ahmed SH Chowdhury TA Hussain S Syed A Karamat A Helmy A . Ramadan and diabetes: a narrative review and practice update. Diabetes Ther. (2020) 11:2477–520. doi: 10.1007/s13300-020-00886-y 32909192 PMC7480213

[5] Loh HH Lim LL Loh HS Yee A . Safety of Ramadan fasting in young patients with type 1 diabetes: a systematic review and meta-analysis. J Diabetes Investig. (2019) 10:1490–501. doi: 10.1111/jdi.13054 30938074 PMC6825934

[6] Almalki M AlSaeed AA AlNomi AA AlSufyani M Albedaiwi K Alshahrani F . Validity of the new International Diabetes Federation-Diabetes and Ramadan (IDF-DAR) risk stratification score and fasting experience of Saudi patients with diabetes during Ramadan: insights from a cross-sectional study. Cureus. (2025) 17:e79351. doi: 10.7759/cureus.79351 40125211 PMC11929123

[7] Kadiki OA . Diabetes mellitus and ramadan. Garyounis Med J. (1989) 12:32–4.

[8] Burge MR Garcia N Qualls CR SChade DS . Differential effects of fasting and dehydration in the pathogenesis of diabetic ketoacidosis. Metabolism. (2001) 50:171–7. doi: 10.1053/meta.2001.20194 11229425

[9] Kitabchi AE Umpierrez GE Miles JM Fisher JN . Hyperglycemic crises in adult patients with diabetes. Diabetes Care. (2009) 32:1335–43. doi: 10.2337/dc09-9032 19564476 PMC2699725

[10] Alshahrani M Alraddadi A . Incidence of diabetic ketoacidosis during Ramadan compared with non-fasting months in King Saud Medical City, Riyadh, Saudi Arabia. J Family Med Prim Care. (2022) 11:3905–8. doi: 10.4103/jfmpc.jfmpc_2004_21 36387634 PMC9648314

[11] AlZahrani AM Zawawi MM Almutairi NA Alansari AY Bargawi AA . The impact of Ramadan on visits related to diabetes emergencies at a tertiary care center. BMC Emerg Med. (2021) 21:162. doi: 10.1186/s12873-021-00555-8 34949164 PMC8705188

[12] Beshyah AS Beshyah SA . The incidence of diabetic ketoacidosis during Ramadan fasting: a 10-year single-centre retrospective study. Diabetes Res Clin Pract. (2019) 150:296–300. doi: 10.1016/j.diabres.2019.01.018 30685349

[13] Abdelgadir EI Hafidh K Basheir AM Afandi BO Alawadi F Rashid F . Comparison of incidences, hospital stay, and precipitating factors of diabetic ketoacidosis in Ramadan and the following month in three major hospitals in the United Arab Emirates: a prospective observational study. J Diabetes Metab. (2015) 6:2. doi: 10.4172/2155-6156.1000514

[14] Abdelgadir EI Hassanein MM Bashier AM Abdelaziz S Baki S Chadli A . A prospective multi-country observational trial to compare the incidences of diabetic ketoacidosis in the month of Ramadan, the preceding month, and the following month (DKAR international). J Diabetes Metab Disord. (2016) 15:1–6. doi: 10.1186/s40200-016-0272-4 27826544 PMC5097841

[15] Elmehdawi R Ehmida M Elmagrehi H . Incidence of diabetic ketoacidosis during Ramadan fasting in Benghazi-Libya. Oman Med J. (2009) 24:99. doi: 10.5001/omj.2009.23 PMC3273945 22334853

[16] Tong CV Yow HY Noor NM Hussein Z . Diabetes emergencies around Ramadan study (DEARS): a multicenter study of diabetes emergencies admitted before, during, and after Ramadan in Malaysia. Diabetes Res Clin Pract. (2021) 175:108854. doi: 10.1016/j.diabres.2021.108854 33961901

[17] Alabbood MH Ho KW Simons MR . The effect of Ramadan fasting on glycaemic control in insulin-dependent diabetic patients: a literature review. Diabetes Metab Syndr. (2017) 11:83–7. doi: 10.1016/j.dsx.2016.06.028 27402028

[18] Beshyah SA Chowdhury TA Ghouri N Lakhdar AA . Risk of diabetic ketoacidosis during Ramadan fasting: a critical reappraisal. Diabetes Res Clin Pract. (2019) 151:290–8. doi: 10.1016/j.diabres.2019.02.027 30836132

[19] Gandhi A Jeun R Wang Z Khan S Best C Lavis V . Etiologies and outcomes of diabetic ketoacidosis in cancer patients: a retrospective analysis. Cancers (Basel). (2025) 17:2728. doi: 10.3390/cancers17172728 40940823 PMC12427185

[20] Alourfi Z Homsi H . Precipitating factors, outcomes, and recurrence of diabetic ketoacidosis at a university hospital in Damascus. Avicenna J Med. (2015) 5:11–5. doi: 10.4103/2231-0770.148503 25625084 PMC4296391

[21] Addisu ZD Demsie DG Beyene DA Tafere C . Prevalence of in-hospital mortality among adult patients with diabetic ketoacidosis in Ethiopia: a systematic review and meta-analysis of observational studies. Front Clin Diabetes Healthc. (2025) 6:1501167. doi: 10.3389/fcdhc.2025.1501167 40265138 PMC12011865

[22] Albuali WH Al-Qahtani MH . Diabetic ketoacidosis and its severity predictors in type 1 diabetic children: a 10-year experience of a teaching hospital in Saudi Arabia. Rev Diabetes Stud. (2022) 18:146–51. doi: 10.1900/RDS.2022.18.146 36309773 PMC9652709

[23] Alhamdani YF Almadfaa LO AlAgha AE . Clinical variables influencing the severity of diabetes ketoacidosis. Saudi Med J. (2024) 45:502–9. doi: 10.15537/smj.2024.45.4.20240058 38734437 PMC11147548

[24] Rashid MO Sheikh A Salam A Farooq S Kiran Z Islam N . Diabetic ketoacidosis characteristics and differences in type 1 versus type 2 diabetes patients. J Ayub Med Coll Abbottabad. (2017) 29:398–402. 29076669

[25] Almazrouei R Siddiqua AR Alnuaimi M Al-Shamsi S Govender R . Clinical and biochemical characteristics of diabetic ketoacidosis in adults with type 1 or type 2 diabetes at a tertiary hospital in the United Arab Emirates. Front Clin Diabetes Healthc. (2022) 3:918253. doi: 10.3389/fcdhc.2022.918253 36992724 PMC10012054

[26] Vitale RJ Card CE Lichtman JH Weyman K Michaud C Sikes K . An effective diabetic ketoacidosis prevention intervention in children with type 1 diabetes. SAGE Open Nurs. (2018) 4:2377960818804742. doi: 10.1177/2377960818804742 33415207 PMC7774356

[27] Alawadi F Alsaeed M Bachet F Bashier A Abdulla K Abuelkheir S . Impact of provision of optimum diabetes care on the safety of fasting in Ramadan in adult and adolescent patients with type 1 diabetes mellitus. Diabetes Res Clin Pract. (2020) 169:108466. doi: 10.1016/j.diabres.2020.108466 32971155

[28] Sperkowska BM Chrustek A Gryn-Rynko A Proszowska A . Dietary interventions for adults with type 1 diabetes: clinical outcomes, guideline alignment, and research gaps—a scoping review. Nutrients. (2025) 17:3349. doi: 10.3390/nu17213349 41228423 PMC12609816

[29] Liao J Wang T Li Z Xie H Wang S . Experiences and views of people with diabetes during Ramadan fasting: a qualitative meta-synthesis. PloS One. (2020) 15:e0242111. doi: 10.1371/journal.pone.0242111 33226993 PMC7682869

