# Supplementary material for: The proportion of diabetic ketoacidosis emergencies during Ramadan fasting: a retrospective study
Source: Front Endocrinol (Lausanne). 2026 Aug 31;17:1895364. doi: 10.3389/fendo.2026.1895364 (PMC13572981; doi:10.3389/fendo.2026.1895364)
Supplement: Supplementary file 1 [file DataSheet1.docx]

The proportion of diabetic ketoacidosis emergencies during Ramadan fasting, Retrospective study

Authors: Maram Hassan AlSufyani, Nawal K Alanazi, Areej Yahya Hamdi, Reema Alalmaee, Gadah Aljarallah, Rahaf Alsufyani, Ahmad Sumaily

# Abstract

**Objectives:** Ramadan fasting is an annual religious event of the Muslim calendar that was shown to impact diabetes control. Relationship between Ramadan fasting and increased diabetic ketoacidosis (DKA) presentation was studied in several settings but results are still mixed. Our study aims at enriching the knowledge gap and estimate frequency and correlates of DKA emergencies during Ramadan month using a more informative study design with repeated observations over three months during the three year-study period.

**Methods:** Retrospective medical record-based observational 3-year study with repeated observations over the three-month period of pre-Ramadan, Ramadan, and post-Ramadan.

**Results:** This study included a total of (n = 125) patients with an established diagnosis of DKA who presented to the hospital during the 3-year study period. Of whom there were (n = 56, 44.8%) patients who were diagnosed with DKA during Ramadan, far in excess of other months by a 1.75 ratio for Shabaan and a 1.51 ratio for Shawwal. Severity of DKA presentations were far less in Ramadan than other months (odds reduced by 47.4%, p = 0.01803). Also, older age was associated with more severe presentation (odds increased by 4.7% for every one older year, p = 0.00885).

**Conclusions:** Consistent with literature, we found that severity of DKA during Ramadan was likely to occur in older adults. Also, we uncovered that when a DKA is diagnosed in Ramadan it is less likely to be severe than both Shabaan and Shawwal months presentations combined. This, we theorize, may be explained by the higher carbohydrate dietary content during Iftar (breakfast) and Sahoor (pre-fasting meal). This is not a well-stated solid fact though and should wait for studies that focus on the association between nutritional intake during these special times and severity of DKA presentations.

**Keywords:** Diabetic ketoacidosis; hyperglycemia, Diabetic emergency, correlates, Ramadan.

**Supplementary material 1**

Figure 1

Figure 2

**Supplementary material 2**

Table 25: Frequency of precipitating factors for DKA

| Precipitating factor | count | % |
| --- | --- | --- |
| "AKI" | 2 | 1.6% |
| "Community Acquired Pneumonia" | 1 | 0.8% |
| "Epigastric Pain" | 3 | 2.4% |
| "Infection" | 2 | 1.6% |
| "Insulin Pump Malfunction" | 1 | 0.8% |
| "Newly Diagnosed" | 11 | 8.8% |
| "Non-compliance" | 98 | 78.4% |
| "Alcohol" | 1 | 0.8% |
| "Pregnancy" | 1 | 0.8% |
| "URTI" | 5 | 4% |
| "Pancreatitis" | 1 | 0.8% |
| "UTI" | 1 | 0.8% |
| "Gastroenteritis" | 2 | 1.6% |

Table 36: Frequency of symptomatology duration prior to DKA presentation

| Duration (days) | 1 | 2 | 3 | 4 | 7 | 10 |
| --- | --- | --- | --- | --- | --- | --- |
| Count (patients) | 83 | 25 | 10 | 3 | 1 | 1 |
| Proportion | 66.4% | 20% | 8% | 2.4% | 0.8% | 0.8% |

Table 47: Hospitalization in days folllowing DKA presentation

| Day of discharge | 1 | 2 | 3 | 4 | 5 | 6 | 9 | 11 | 13 |
| --- | --- | --- | --- | --- | --- | --- | --- | --- | --- |
| Count of patients | 44 | 42 | 24 | 5 | 1 | 5 | 1 | 1 | 1 |
| Proportion of patients | 35.2% | 33.6% | 19.2% | 4% | 0.8% | 4% | 0.8% | 0.8% | 0.8% |

Logistic regression results for

Demographic/clinical factors

| Factor | β | SE [β] | Z value | P value |
| --- | --- | --- | --- | --- |
| Age | 0.063310 | 0.047181 | 1.342 | 0.1796 |
| Gender: Male | -1.679367 | 0.716462 | -2.344 | 0.0191 |
| Diabetes duration | 0.003962 | 0.005434 | 0.729 | 0.4659 |
| Symptoms duration | -0.630478 | 0.417577 | -1.510 | 0.1311 |
| Acidosis duration | -0.020752 | 0.032901 | -0.631 | 0.5282 |
| Hospitalization duration | -0.052970 | 0.164369 | -0.322 | 0.7473 |
| PreRamadan education | -0.043548 | 0.793272 | -0.055 | 0.9562 |
| HgA1c | 0.068247 | 0.131845 | 0.518 | 0.6047 |

Laboratory factors

| Factor | β | SE [β] | Z value | P value |
| --- | --- | --- | --- | --- |
| Urine ketones | -0.011414 | 0.006177 | -1.848 | 0.0646 . |
| pH | -3.534671 | 3.256383 | -1.085 | 0.2777 |
| Bicarbonate | 0.052900 | 0.075386 | 0.702 | 0.4828 |
| Potassium | 0.161478 | 0.238847 | 0.676 | 0.4990 |
| Pulse | -0.009157 | 0.015328 | -0.597 | 0.5502 |
| Respiratory rate | -0.023457 | 0.056893 | -0.412 | 0.6801 |
| Systolic BP | -0.001339 | 0.018021 | -0.074 | 0.9408 |
| Diastolic BP | -0.014038 | 0.022860 | -0.614 | 0.5391 |
| Temperature | 0.306448 | 0.966767 | 0.317 | 0.7513 |
| Oxygen | 0.120674 | 0.173189 | 0.697 | 0.4859 |
| BMI | 0.045106 | 0.039673 | 1.137 | 0.2556 |
| Sodium | -0.01500 | 0.04548 | -0.330 | 0.742 |

Precipitating factors

| Factor | β | SE [β] | Z value | P value |
| --- | --- | --- | --- | --- |
| AKI | 16.80338 | 2375.15462 | 0.007 | 0.994 |
| Community-acquired penumonia | -17.36833 | 3956.18041 | -0.004 | 0.996 |
| Epigastric pain | -17.35844 | 2282.42362 | -0.008 | 0.994 |
| Infection | 16.78838 | 2375.15462 | 0.007 | 0.994 |
| Bump malfunction | -17.38333 | 3956.18041 | -0.004 | 0.996 |
| Newly diagnosed | -0.34469 | 1.02665 | -0.336 | 0.737 |
| Non-compliance | 0.05915 | 0.82866 | 0.071 | 0.943 |
| Alcohol | -17.39748 | 3956.18033 | -0.004 | 0.996 |
| URTI | -0.19844 | 0.98030 | -0.202 | 0.840 |
| Panceriatitis | 17.50879 | 3956.18050 | 0.004 | 0.996 |
| UTI | 17.79381 | 3956.18041 | 0.004 | 0.996 |
| Gastroenteritis | 18.24995 | 2756.07 | 0.007 | 0.995 |
